# The role of the entorhinal cortex in epileptiform activities of the hippocampus

**DOI:** 10.1186/1742-4682-11-14

**Published:** 2014-03-24

**Authors:** Hui Ren, Ye-Jun Shi, Qin-Chi Lu, Pei-Ji Liang, Pu-Ming Zhang

**Affiliations:** 1 School of Biomedical Engineering, Shanghai Jiao Tong University, Shanghai 200240, China; 2 Department of Neurology, Ren Ji Hospital, School of Medicine, Shanghai Jiao Tong University, Shanghai 200127, China

**Keywords:** Computational model, Hippocampal CA3 region, Entorhinal cortex, Valproate, Temporal lobe epilepsy

## Abstract

**Background:**

Temporal lobe epilepsy (TLE) is the commonest type of epilepsy in adults, and the hippocampus is indicated to have a close relationship with TLE. Recent researches also indicate that the entorhinal cortex (EC) is involved in epilepsy. To explore the essential role that the EC may play in epilepsy, a computational model of the hippocampal CA3 region was built, which consisted of pyramidal cells and two types of interneurons. By changing the input signals from the EC, the effects of EC on epileptiform activities of the hippocampus were investigated. Additionally, recent studies have found that the antiepileptic drug valproate (VPA) can block ictal discharges but cannot block interictal discharges in vitro, and the mechanism under this phenomenon is still confusing. In our model, the effects of VPA on epileptiform activities were simulated and some mechanisms were explored.

**Results:**

Interictal discharges were induced in the model without the input signals from the EC, whereas the model with the EC input produced ictal discharges when the EC input contained ictal discharges. The GABA-ergic connection strength was enhanced and the NMDA-ergic connection strength was reduced to simulate the effects of VPA, and the simulation results showed that the disappearance of ictal discharges in the model mainly due to the disappearance of ictal discharges in the input signals from the EC.

**Conclusions:**

Simulation results showed that ictal discharges in the EC were necessary for the hippocampus to generate ictal discharges, and VPA might block the ictal discharges in the EC, which led to the disappearance of ictal discharges in the hippocampus.

## Introduction

Temporal lobe epilepsy (TLE) is the commonest type of epilepsy in adults, and about 75% of patients with mesial TLE are considered to have drug-resistant epilepsy [1]. It has been commonly accepted that the hippocampus has a close relationship with the generation of TLE. In specimens from surgical resections and post-mortem studies of patients with TLE, neuronal loss, atrophy, and gliosis have been revealed in the hippocampus [2]. It has been reported that epileptiform activities depend crucially on intrinsic neuronal properties, and the organisation of the synaptic networks [3]. The hippocampus has massive recurrent excitatory connections, intrinsically burst-generating cells, as well as closely spaced cell bodies and dendrites, and these all make it easy to generate epileptiform activities [4,5].

In spite of the essential role that the hippocampus plays in the generation of epileptiform activities, observations in animal models also have indicated that the epileptogenic zone is broad, and some other limbic regions may be involved in the generation of TLE as well [2]. Now, many scientists have shown much interest in the role the entorhinal cortex (EC) plays in epilepsy [6]. Magnetic resonance imaging studies showed that the EC was damaged in patients with TLE [7], and observations in both rats and patients with TLE displayed preferential neuronal loss in the EC [8,9]. There exist extensive reciprocal connections between the EC, hippocampus, and other brain areas, which make the EC a potential candidate for generating and propagating TLE seizures [10].

Epileptiform activities appear as interictal discharges and ictal discharges. Interictal discharges are the simplest identifiable epileptiform activities, which last tens or hundreds of milliseconds [4], and ictal discharges (also termed seizures) represent the critical events and the primary clinical burden of an active epileptic condition, which usually last more than 10 seconds [4,11]. It has been suggested that the EC may contribute to the initiation of ictal discharges [6,12,13]. It was reported that cutting the perforant pathway blocked the ictal discharges in the hippocampus, but not in the EC, where ictal discharges continued to occur with similar features compared to those seen in the combined EC-hippocampal slices where the perforant pathway was preserved [6,14]. Our laboratory has done researches about epileptiform activities induced by Mg^2+^-free artificial cerebrospinal fluid (ACSF), which unblocks the N-methyl-D-aspartate (NMDA) receptors, in hippocampal slices and combined EC-hippocampal slices as well [15-19]. We found that ictal discharges were only induced in the combined EC-hippocampal slices, which indicated that the EC was very essential for the hippocampus to initiate ictal discharges [18,19]. Although the EC has been shown to have a close relationship with the generation of epileptiform activities, the exact role it plays in epilepsy is still unclear.

Many experimental studies in hippocampal slices indicated that the CA3 field was the place that could generate interictal activities [6,20]. Pyramidal cells in the CA3 receive signals from the dentate gyrus (DG) via mossy fibers, and from the EC via the perforant pathway, and they project to the CA1 [21]. Divergent connections of the CA3 pyramidal cells with local cells and cells of the other fields allow for the expansion of synchronous population discharges [20]. Many models of the CA3 field had been built to investigate the mechanisms of different epileptiform activities, such as carbachol-driven rhythmic population oscillations [22], picrotoxin-induced synchronized after-discharges [23], or low-Mg^2+^ induced neuronal bursts and after-discharges [24]. But they only simulated interictal discharges and didn’t consider the impacts that the EC may make on epileptiform activities of the hippocampus.

Valproate (VPA) is one of the major antiepileptic drugs (AEDs) used today, and its ability to control epileptiform activities mainly depends on enhancing the GABA-ergic inhibitory functions and reducing the NMDA-ergic excitatory functions of the nervous system [25]. Researchers have found that VPA could block ictal discharges but couldn’t completely block interictal discharges in vitro [26-28]. In our experiments, we also found that the application of 3 mM VPA suppressed the frequency of interictal discharges and completely blocked the ictal discharges in the combined EC-hippocampal slices [19]. However, the mechanism of this phenomenon is still confusing.

In this work, we built a model of the hippocampal CA3 region to simulate interictal and ictal discharges based on NEURON [29]. The model contained 4 neurons including 2 pyramidal cells, one basket cell and one oriens-lacunosum molecular (OLM) cell. The model contained 2 inputs which were from the DG and EC respectively. Simulation results indicated that interictal discharges were induced in the model without the EC input, whereas the model with the EC input produced ictal discharges when the EC input contained ictal discharges. From the results, we supposed that ictal discharges from the EC were necessary for the hippocampus to generate ictal discharges. In our model, the GABA-ergic inhibitory connections were enhanced and the NMDA-ergic excitatory connections were reduced to simulate the effects of VPA. Simulation results showed that the disappearance of ictal discharges in the model mainly due to the disappearance of ictal discharges of the input signals from the EC. Thus, we supposed that VPA might block the ictal discharges in the EC of combined EC-hippocampal slices, which led to the disappearance of ictal discharges in the hippocampus.

## Results

### Simulation reproduces Mg^2+^-free-ACSF induced interictal discharges

**Figure 1:**
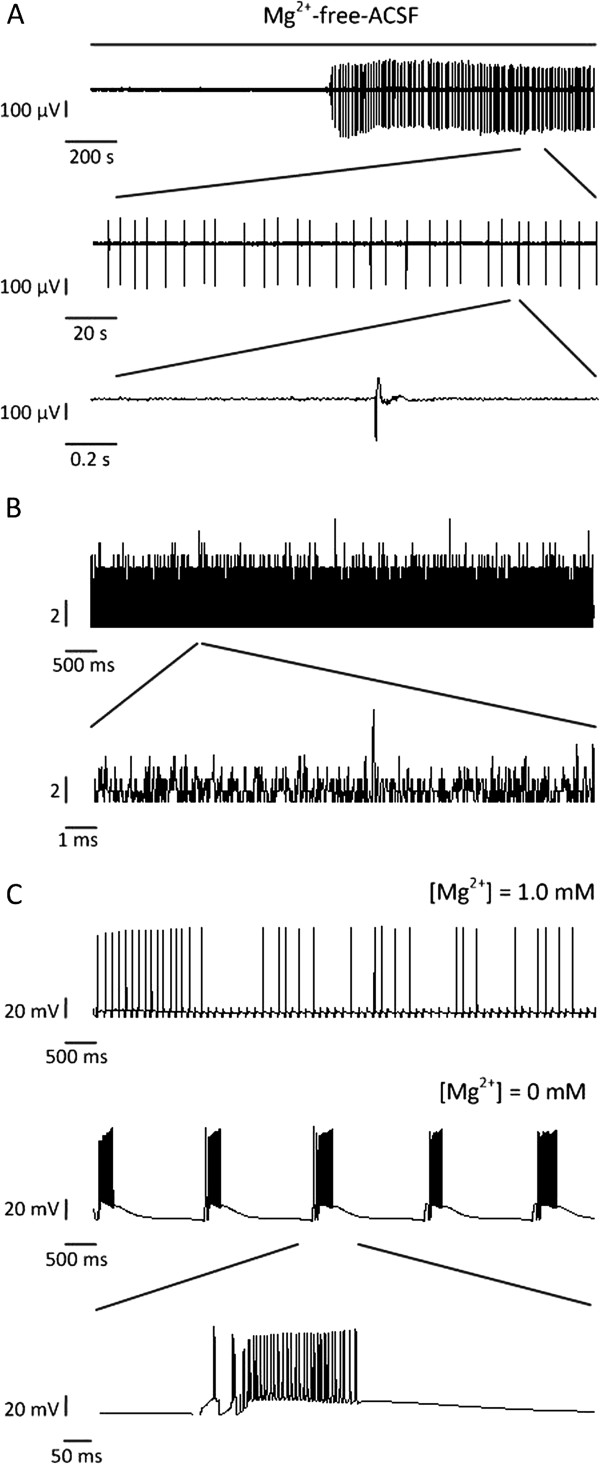
**Interictal discharges in experiments and in the model, and input signals from DG. (A)** LFPs in CA3 region recorded by MEA in a hippocampal slice. Interictal discharges occurred with more than 10 minutes’ delays after Mg^2+^-free-ACSF perfusion. **(B)** Simulated input signals from the DG which followed a Poisson process (λ = 1). **(C)** Somatic membrane potentials of one pyramidal cell under different Mg^2+^ concentrations in the model without the input from the EC. Interictal discharges occurred when [Mg^2+^] was 0 mM. [Mg^2+^]: extracellular Mg^2+^ concentration.

In our experiments, the application of Mg^2+^-free-ACSF consistently induced interictal discharges in adult rat hippocampal slices [15-17] and adult mouse hippocampal slices [18,19]. Figure 1A shows an example of Mg^2+^-free-ACSF induced interictal discharges in one mouse hippocampal slice. These signals were local field potentials (LFPs) recorded by one electrode of the micro-electrode array (MEA) located in the CA3 region. The onset of interictal discharges in different slices appeared with different time delays after Mg^2+^-free-ACSF perfusion began, ranging between 10 and 20 min (15.8 ± 2.6 min, n = 4 slices) [18]. Signals measured in the model were the membrane potentials of pyramidal cells, which seemed similar to the intracellular recordings and different from LFPs recorded in experiments as shown in Figure 1A. LFPs were the electric potentials recorded in the extracellular space and it had been shown that LFPs were synchronized with intracellular recordings in the same region during epileptiform activities [27,30].

As the hippocampal slices didn’t contain EC, we only considered the input from the DG, which followed a Poisson process (λ = 1) [31] in this model (Figure 1B). The somatic membrane potentials of one pyramidal cell under different Mg^2+^ concentrations were plotted in Figure 1C. When the Mg^2+^ concentration was 1.0 mM, which was the normal Mg^2+^ concentration in the extracellular solution [32], interictal discharges were absent. However, when the Mg^2+^ concentration was changed to 0 mM, interictal discharges occurred. Every single burst of interictal discharges consisted of a train of several spikes riding on a large depolarizing wave, which was similar to the paroxysmal depolarizing shift recorded in experiments [4,33]. What’s more, the interictal discharges in the simulation occurred when the input signals followed a Poisson process, which supported the viewpoint, indicated in many experimental studies, that the CA3 field could generate interictal activities [6,16,18-20].

### Ictal and interictal discharges induced by the EC input

**Figure 2:**
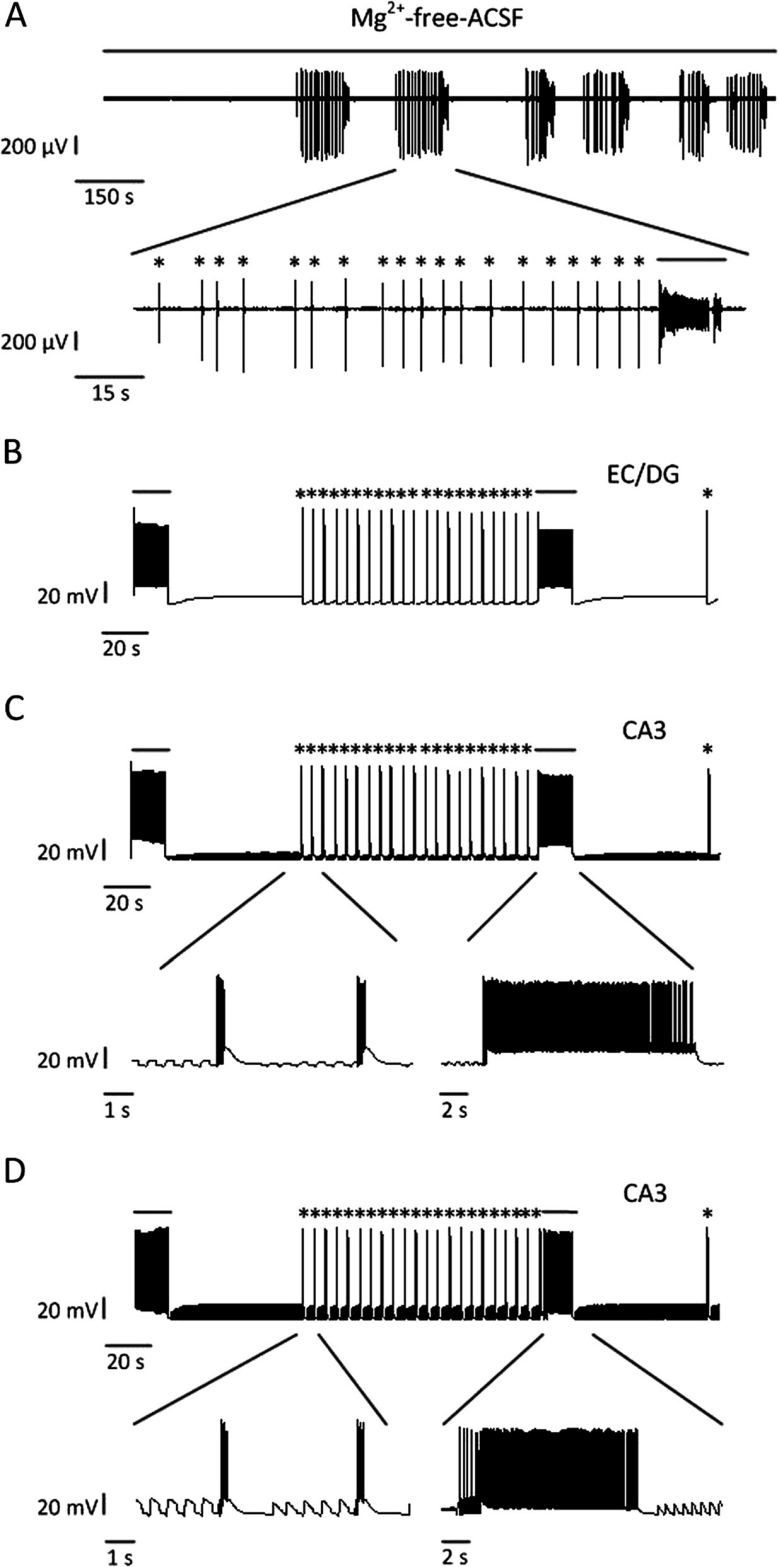
**Interictal (asterisks) and ictal (continuous lines) discharges in experiments and the model, and input signals. (A)** LFPs in the CA3 region recorded by MEA in a combined EC-hippocampal slice. Interictal discharges occurred regularly before an ictal discharge. **(B)** Simulated input signals from the DG or EC, which were alternate interictal and ictal discharges. **(C)** Somatic membrane potentials of one pyramidal cell in the model with the input from the EC. When input signals from the DG and EC were alternate interictal and ictal discharges as shown in **(B)**, pyramidal cells in the model produced interictal and ictal discharges. **(D)** Somatic membrane potentials of one pyramidal cell in the model with the input from the EC. When the input signals from the DG followed a Poisson process as shown in Figure 1B and the input signals from the EC were alternate interictal and ictal discharges as shown in **(B)**, pyramidal cells in the model produced interictal and ictal discharges.

In our experiments, two types of epileptiform discharges were recorded in the mouse combined EC-hippocampal slices during the application of Mg^2+^-free-ACSF (Figure 2A). Interictal discharges occurred regularly in the CA3 region, and they generally appeared before an ictal discharge with a frequency of 0.22 ± 0.06 Hz, and the frequency of ictal discharges was about 0.004 ± 0.001 Hz (n = 4 slices) [18]. The epileptiform activities measured in the DG and EC were synchronized with those measured in the CA3 region [18,19,34]. Also, according to experimental studies, the EC was indicated to be the first site to produce ictal discharges in combined EC-hippocampal slices [6,18,19]. In this model, interictal and ictal discharges were added into the network to simulate inputs from the EC and DG (Figure 2B). As a result, the pyramidal cells in the model produced interictal and ictal discharges (Figure 2C). The interictal discharges appeared before an ictal discharge with a frequency of 0.2 Hz and the ictal discharges appeared with a frequency of 0.005 Hz. These patterns were synchronized with the input signals, which supported the viewpoint that synchronized firing occurred during Mg^2+^-free-ACSF induced epileptiform activities [24].

In the simulation, ictal discharges occurred when the input signals from the EC and DG contained ictal activities, and there were no reports or experimental results showing that the DG was the first place to generate ictal discharges. We supposed that ictal discharges in the hippocampus were induced by the EC. To confirm this supposition, we set the input from the DG as signals followed a Poisson process (λ = 1) as shown in Figure 1B, and didn’t change the input from the EC (Figure 2B). The simulation result showed that ictal discharges still occurred (Figure 2D). In this way, we deduced that the EC induced the hippocampus to generate ictal discharges in combined EC-hippocampal slices.

### Effects of VPA on the epileptiform activities

**Figure 3:**
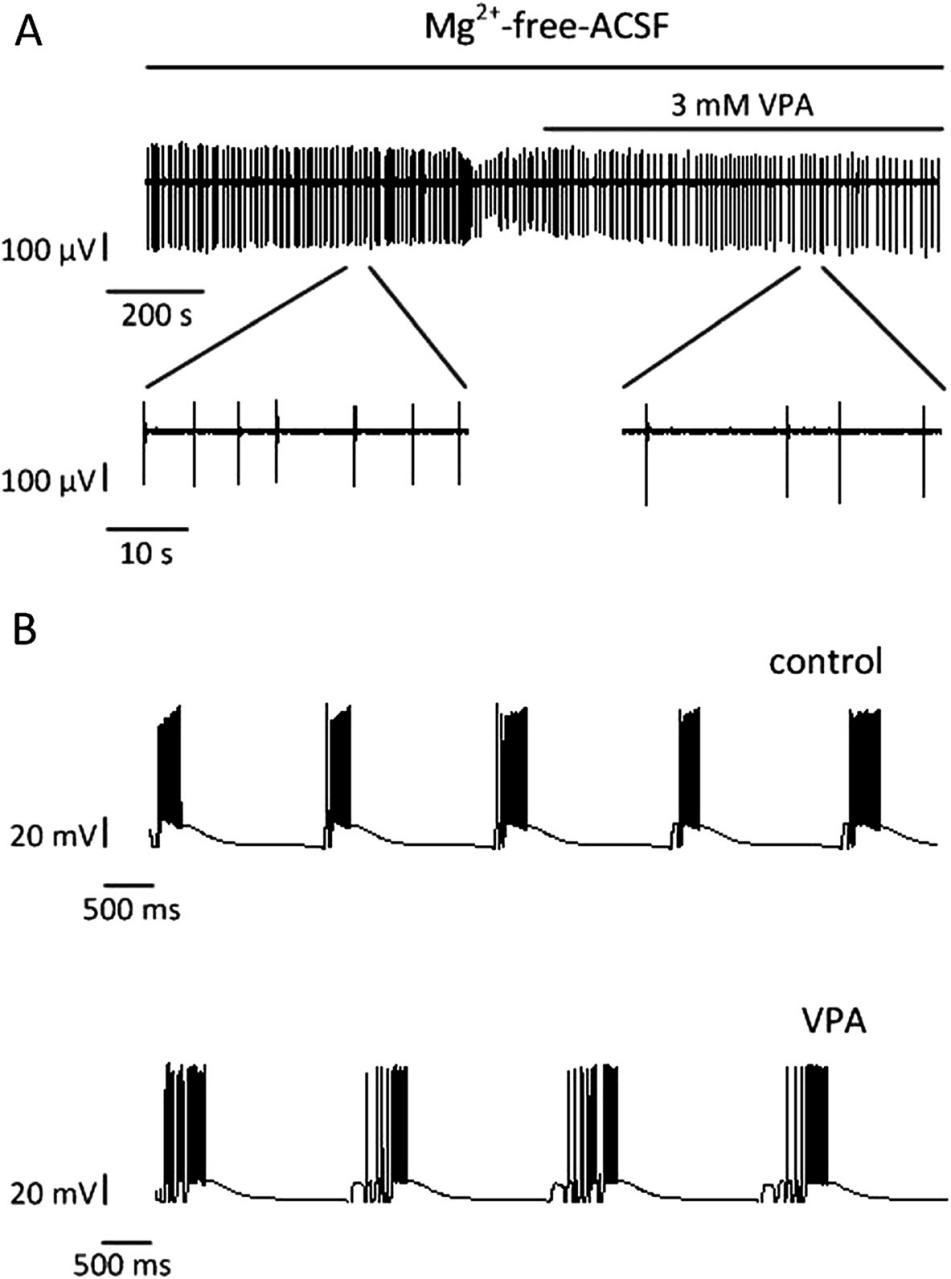
**Effects of VPA on interictal activities in experiments and simulations. (A)** Interictal discharges before and during 3 mM VPA application in one hippocampal slice, which represents the recording of one electrode in the CA3 region. **(B)** The somatic membrane potentials of one pyramidal cell in the model without the EC input. The input signals from the DG followed a Poisson process as shown in Figure 1B. When the GABA-ergic connection strength was enhanced and the NMDA-ergic connection strength was reduced, the frequency of interictal discharges decreased.

In our experiments, the application of 3 mM VPA suppressed the frequency of interictal discharges induced by Mg^2+^-free-ACSF, but could not completely block them in the mouse hippocampal slices [19], and one example was shown in Figure 3A. In the model without the input from the EC, the GABA-ergic inhibitory connection strength was enhanced and the NMDA-ergic excitatory connection strength was reduced to simulate the effects of VPA on interictal activities. Figure 3B shows one example of the membrane potentials of one pyramidal cell when the GABA-ergic connection strength is tripled and the NMDA-ergic connection strength is reduced by 20% in the model. We found that the frequency of interictal discharges decreased about 22.2%.

**Figure 4:**
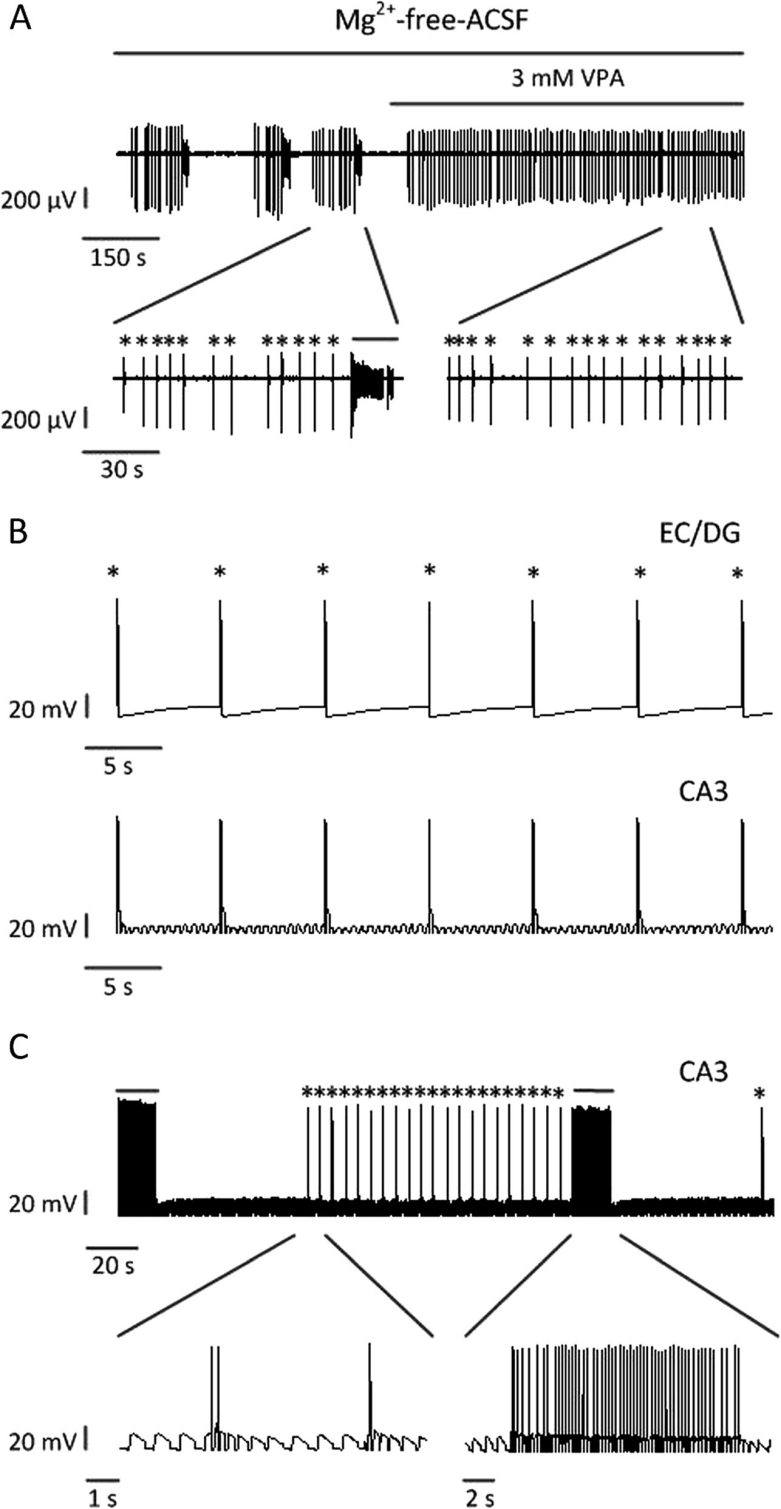
**Effects of VPA on interictal (asterisks) and ictal (continuous lines) activities in experiments and simulations. (A)** Epileptiform discharges before and during 3 mM VPA application in a combined EC-hippocampal slice, which represents the recordings of one electrode in the CA3 region. **(B)** The somatic membrane potentials of a pyramidal cell in the model with the input from the EC. When the GABA-ergic connection strength was enhanced and the NMDA-ergic connection strength was reduced, and the input signals from the EC and DG were interictal discharges with a lower frequency compared to interictal discharges added previously as shown in Figure 2B, ictal discharges disappeared and interictal discharges still existed. **(C)** The somatic membrane potentials of a pyramidal cell in the model with the input from the EC. When the GABA-ergic connection strength was enhanced and the NMDA-ergic connection strength was reduced, and the input signals from the DG followed a Poisson process as shown in Figure 1B, and the input signals from the EC were alternate interictal and ictal discharges as shown in Figure 2B, ictal and interictal discharges still existed.

According to our experimental studies in the mouse combined EC-hippocampal slices, the application of 3 mM VPA completely blocked ictal discharges, and suppressed but didn’t completely block interictal discharges (Figure 4A). The frequency of interictal discharges changed from 0.22 ± 0.06 Hz to 0.15 ± 0.03 Hz (n = 4 slices) [19]. Suggested by the experimental data, the EC and DG produced interictal discharges with lower frequencies after the application of VPA in combined EC-hippocampal slices [27]. Thus, in the model with the input from the EC, interictal discharges with a frequency of 0.15 Hz were added into the network to simulate input signals from the EC and DG (Figure 4B). The pyramidal cells in the model produced interictal discharges synchronized with the input signals (data not shown). When the GABA-ergic connection strength was enhanced more than 10 times and the NMDA-ergic connection strength was reduced by more than 20%, the interictal discharges still existed and were synchronized with the input signals (Figure 4B). Thus, VPA blocked the ictal discharges but could not block the interictal discharges.

The disappearance of ictal discharges in the model seemed due to the disappearance of ictal discharges in the input signals. In this way, we supposed that VPA might block the ictal discharges in the EC of the combined EC-hippocampal slices, which led to the disappearance of ictal discharges in the hippocampus. To confirm this opinion further, ictal and interictal discharges were added into the model to simulate the input signals from the EC, and the signals followed a Poisson process (λ = 1) were added into the model to simulate the input signals from the DG. The model generated interictal and ictal discharges as shown in Figure 2D. When the GABA-ergic connection strength was enhanced more than 50 times and the NMDA-ergic connection strength was reduced by more than 20%, the ictal and interictal discharges still existed (Figure 4C). The results suggested that VPA could not block ictal discharges in the hippocampus when the EC contained ictal discharges. Thus, we deduced that during the application of VPA in combined EC-hippocampal slices, the disappearance of ictal discharges in the hippocampus might result from the disappearance of ictal discharges in the EC.

## Discussion

In this work, we established a model of the hippocampal CA3 region to characterize the effects of the EC on the hippocampal epileptiform activities. In the model without the input from the EC, interictal discharges were induced by changing the Mg^2+^ concentration to 0 mM, which supported the viewpoint that CA3 field could generate interictal activities [6]. In the model with the input from the EC, which contained interictal and ictal discharges, pyramidal cells of the model generated interictal and ictal discharges. Additionally, when the input signals from the DG followed a Poisson process and the input signals from the EC contained ictal discharges, pyramidal cells of the model generated ictal discharges. From the simulation results, we deduced that the EC was the first place to generate ictal discharges in combined EC-hippocampal slices, which was suggested in many previous experimental studies [6,12-14]. Finally, the GABA-ergic inhibitory connection strength was enhanced and the NMDA-ergic excitatory connection strength was reduced to simulate the effects of VPA. As the ictal discharges of the model disappeared when the input signals from the EC didn’t contain ictal discharges, we supposed that the disappearance of ictal discharges in the hippocampus of combined EC-hippocampal slices might due to the disappearance of ictal discharges in the EC. In this way, we deduced that VPA might block the ictal discharges in the EC of combined EC-hippocampal slices, which led to the disappearance of ictal discharges in the hippocampus.

Many people have built models of the CA3 region to investigate possible mechanisms of epileptiform activities [22-24,35]. Those models were very large and contained at least hundreds of neurons. Some other people built models of the CA3 region to do researches of the other problems such as schizophrenia [31,36,37]. Those models contained more than 10 neurons. Compared to the models built previously, the model built here was much smaller and only contained 4 neurons. Our model contained the pyramidal cell, the basket cell and the OLM cell, which were the commonest neurons in the CA3 model [31,36,37]. Connections between these neurons were based on anatomical study results [21,38]. The model also contained the commonest receptors, which were AMPA, NMDA and GABA_A_ receptors [31,35]. Although our model was small, it could generate epileptiform activities and simulate the effects of VPA. But, this small model has many limits. It has been reported that the epileptiform activities may firstly occur in a small number of pyramidal neurons, and then spread to other pyramidal neurons [23]. With our small model, we cannot simulate the spread of epileptiform activities. Additionally, it has been shown that during different periods of epileptiform activities, the number of pyramidal cells that fire synchronously is different [24]. Since there are only two pyramidal cells in our model, it cannot simulate that phenomenon.

The pyramidal cells built in this model were burst generating cells, as it had been reported that most pyramidal cells in the CA3 field could generate bursts and the generation of epileptiform activities were related to the burst generating cells [4,5,39]. However, the CA3 field also contains nonbursting pyramidal cells, which show action potentials with a property of spike frequency adaption after the application of somatic current injections [21,39]. The discharges of bursting pyramidal cells always precede the population discharges, and bursting pyramidal cells may be the pacemakers of epileptiform activities, whereas nonbursting pyramidal cells only discharge simultaneously with the population discharges during epileptiform activities [40]. So, in our model, we didn’t consider the nonbursting pyramidal cells. But, if we want to research the details of the firing pattern of epileptiform discharges, we may need to consider the nonbursting pyramidal cells.

**Figure 5:**
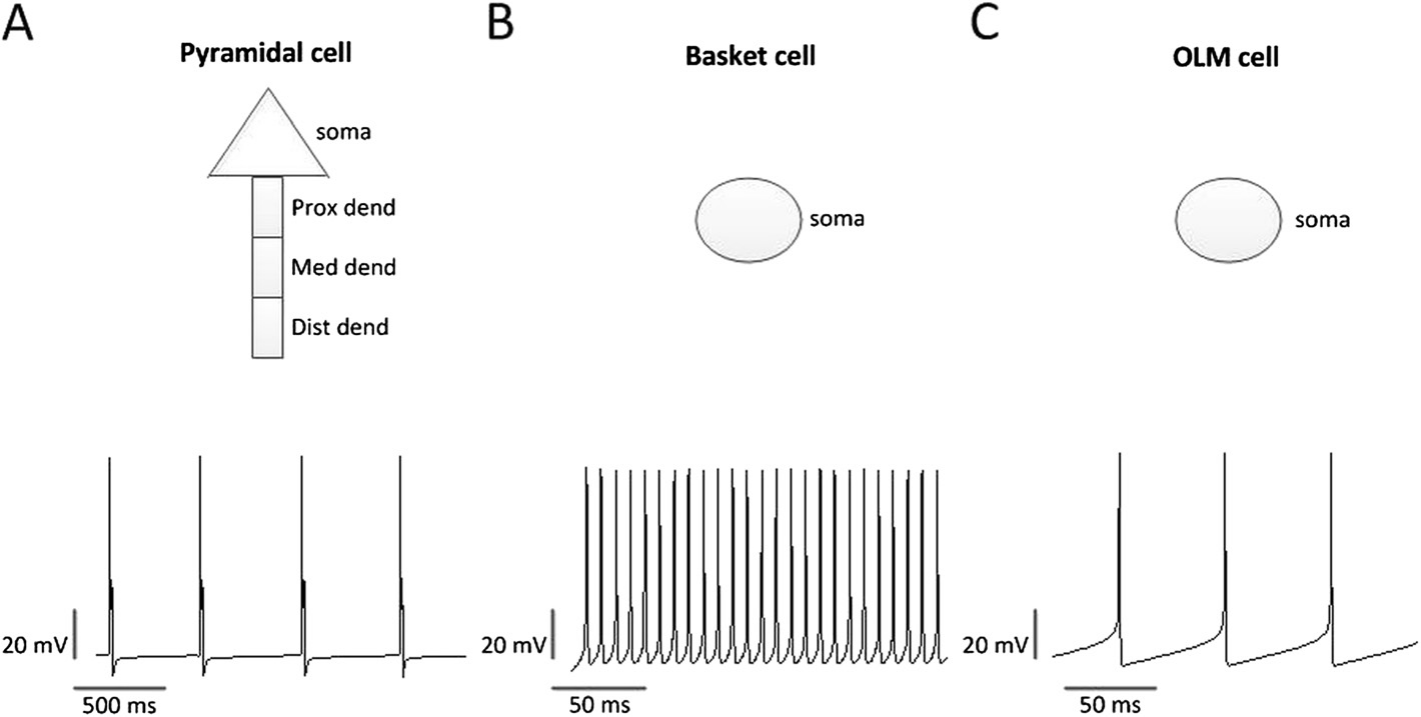
**Structure models for different cell types, and their firing properties in response to current injections. (A)** Structure model of the pyramidal cell and its firing properties in response to 0.23 nA somatic current injections. Prox dend: proximal dendritic compartment; Med dend: medial dendritic compartment; Dist dend: distal dendritic compartment. **(B)** Structure model of the basket cell and its firing properties in response to 0.003 nA somatic current injections. **(C)** Structure model of the OLM cell and its firing properties in response to 0.003 nA somatic current injections.

In normal physiological conditions, the structure of the pyramidal cell is very complex [39], and many people have built different reconstructed CA3 pyramidal cell models. Some models contained more than 200 compartments [39,41], and some contained more than 10 compartments [22-24], or only several compartments [31,36]. The pyramidal cell built in our model consisted of 4 compartments: one somatic and 3 dendritic compartments (Figure 5). Although the structure of this cell model was different from many other models, its responses to the somatic current injections were similar to the firing patterns of pyramidal cells measured in experiments [39]. However, the distribution of ion channels in this cell model could not be the same as that in the cell models whose structures were similar with that of a real pyramidal cell, which made this model’s output have some differences with the real pyramidal cells’ firing activities. If we investigate the effects of the firing pattern or the distribution of some ion channels of pyramidal cells on the epileptiform activities, we may need to build more detailed pyramidal cells in the future work.

There are many types of interneurons in the CA3 field. In this model, we only considered the basket cell and the OLM cell, which were 2 common types in the CA3 field [21,38]. However, there exist some other types of perisomatic-targeting interneurons (such as the axo-axonic cells) and dendritic-targeting interneurons (such as the bistratified cells) in the CA3 field [21]. The acting sites towards pyramidal cells and the firing patterns of these cells are similar to the basket cells or the OLM cells, but there still exist some differences. The axo-axonic cells project to the proximal dendrites of pyramidal cells and the bistratified cells project to the medial dendrites of pyramidal cells [21,38]. So considering these interneurons may make the CA3 model more reliable. However, our model is small and these cells only compose a small part of CA3 interneurons, so we didn’t consider these cells. If we expand the structure of our network, we’d better add these types of interneurons.

VPA is one of the major AEDs used today, and has high efficiency in treating various seizure types such as myoclonic, generalized tonic-clonic seizures and partial seizures [25,42]. In this work, the GABA-ergic inhibitory connection strength was enhanced and the NMDA-ergic excitatory connection strength was reduced to simulate the effects of VPA. However, VPA may also control epileptiform activities by affecting some ion channels. It has been reported that VPA may affect calcium and potassium channels by interfering with calcium entry into the cell and activating the potassium conductance, which lead to the reduction of neuronal excitability [25,43]. VPA may also inhibit sodium channels [44], although this opinion has been questioned [45]. In this model, we didn’t consider the possible effects of VPA on these channels, which might make the simulated effects of VPA on epileptiform activities smaller than VPA’s real effects.

In the future work, our model may be used to do other researches about epileptiform activities, such as the effects of 6-cyano-7-nitroquinoxaline-2,3-dione (CNQX) or bicuculline on the Mg^2+^-free-ACSF induced epileptiform discharges [24]. However, the model built here is very simple compared with what we have known of the CA3 field. To make the model more similar to the real CA3 network, the number of the pyramidal cells, basket cells and OLM cells in the model can be increased, and more cell types and their connectivity can be included in the future work.

## Methods

### Network structure of the CA3 model

**Figure 6:**
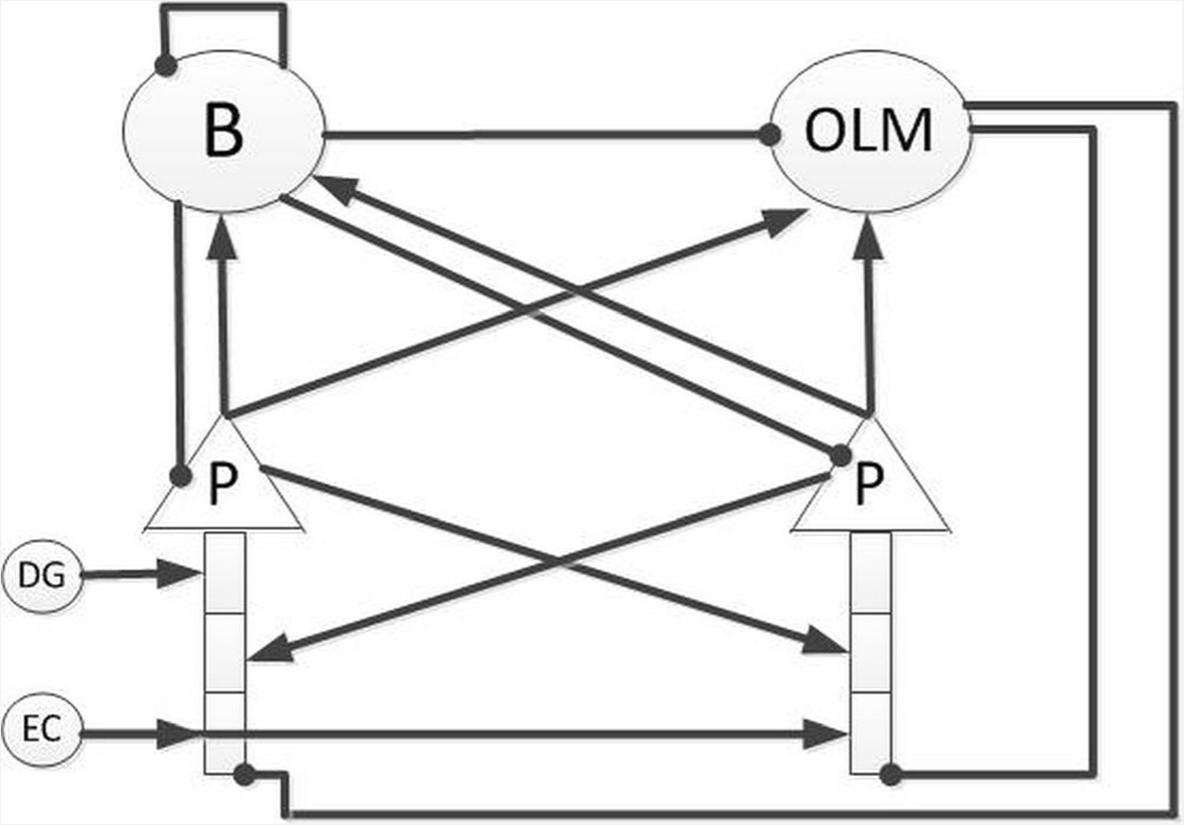
**Simulated CA3 network.** The basket cell (B) inhibits itself, the OLM cell (OLM) and the pyramidal cells (P). The OLM cell inhibits the distal dendrites of the pyramidal cells. Pyramidal cells excite each other and both inhibitory cells. The input from the DG excites the proximal dendrite of one pyramidal cell and the input from the EC excites the distal dendrites of both pyramidal cells. In this diagram, the filled circles represent inhibitory synapses, whereas the arrows represent excitatory synapses.

The network structure of the CA3 model is illustrated in Figure 6. The network consists of 2 pyramidal cells, one OLM cell and one basket cell. Pyramidal cells excite each other, and they also excite the basket cell and OLM cell. The basket cell inhibits itself, the OLM cell and both pyramidal cells. The OLM cell inhibits both pyramidal cells. As shown in Figure 6, the simulated region receives 2 inputs from the EC and DG respectively. The input from the DG excites the proximal dendrite of one pyramidal cell and the input from the EC excites the distal dendrites of both pyramidal cells. Schematic representations of the simulated cells and their responses to current injections are described in Figure 5. The complete mathematical implementation of each cell is described in the Appendix. All simulations were performed using NEURON [29] running on a personal computer under Windows 7.

Basket cells and OLM cells are 2 commonest interneurons in the CA3 models [31,35-37]. It has been reported that interneurons that control the firing activities of pyramidal cells have 2 types: perisomatic-targeting interneurons and dendritic-targeting interneurons. Basket cells belong to the former type and OLM cells belong to the latter [37]. Basket cells control the synchrony of action potentials of pyramidal cells [46]. OLM cells affect the membrane potentials of dendrites of pyramidal cells, where most glutamatergic receptors locate. As the conductance of glutamatergic receptors is related to the local membrane potentials [47,48], OLM cells control the efficacy of glutamatergic inputs [46]. Hence changes in firing patterns of the basket and OLM cells may result in pathological forms of population synchrony, which may lead to epilepsy [46].

### Pyramidal cell

Each simulated pyramidal cell consists of 4 compartments: one somatic and 3 dendritic compartments [36]. Each pyramidal cell contains leak current, sodium current, delayed rectifier potassium (K^+^) current, calcium (Ca^2+^) activated K^+^ current, afterhyperpolarization (AHP) K^+^ current, M-type K^+^ current, L-type Ca^2+^ current, N-type Ca^2+^ current, and T-type Ca^2+^ current [24,31,39,41]. The leak, sodium and delayed rectifier K^+^ currents allow cells to generate action potentials [31]. The Ca^2+^-activated K^+^ current and AHP K^+^ current make the cells have the property of spike-frequency adaptation, which has been observed in most pyramidal cells [49]. The M-type K^+^ current plays an important role in the regulation of firing rate, and it has been reported that small changes of the M current seem to be sufficient to cause epileptic seizures [50]. Voltage-sensitive Ca^2+^ currents may contribute to epileptogenesis and the N-type and L-type Ca^2+^ current have been revealed to regulate a number of neuronal processes, including Ca^2+^-dependent K^+^ currents [51]. In addition, the T-type Ca^2+^ current can control the membrane potentials and intracellular Ca^2+^ concentrations [52]. The parameters of all ionic conductance used in the model are listed in Table 1. Sodium, delayed rectifier K^+^, N-type Ca^2+^ and T-type Ca^2+^ conductance is uniformly distributed throughout the entire neuron, Ca^2+^-activated K^+^ conductance decreases with distance from the soma, AHP K^+^ conductance is lower in distal than in proximal dendrites, and T-type Ca^2+^ conductance exists only in the soma and proximal dendrites. The distribution of the ionic conductance is similar with the parameters of a bursting model of the CA3 pyramidal cell built by Lazarewicz et al. [41]. Responses of the pyramidal cell to current injections are shown in Figure 5A. We can see that the pyramidal cell generates bursts, which have been revealed to be the ability most pyramidal cells in the CA3 region have during the application of somatic current injections [4,5]. In addition, the firing pattern of this pyramidal cell is similar to the pattern measured in experiments [39].

**Table 1:** Passive parameters and ionic conductance of channels for all compartments of pyramidal cells

**Mechanism**	**Soma**	**Prox dend**	**Med dend**	**Dist dend**
C_m,_ μF/cm^2^	1	2	2	2
R_m,_ Ωcm^2^	60000	30000	30000	30000
Ra_,_ Ωcm	200	200	200	200
Leak conductance [S/cm^2^]	0.000017	0.000033	0.000033	0.000033
Sodium conductance [S/cm^2^]	0.035	0.035	0.035	0.035
Delayed rectifier K^+^ conductance [S/cm^2^]	0.005	0.005	0.005	0.005
Ca^2+^-activated K^+^ conductance [S/cm^2^]	0.003	0.00225	0.00075	–
AHP K^+^ conductance [S/cm^2^]	0.0009	–	0.000225	0.000225
M-type K^+^ conductance [S/cm^2^]	0	0.0023	0.00023	0.000023
L-type Ca^2+^ conductance [S/cm^2^]	0.0014	0.0014	–	–
N-type Ca^2+^ conductance [S/cm^2^]	0.0016	0.0016	0.0016	0.0016
T-type Ca^2+^ conductance [S/cm^2^]	0.0005	0.0005	0.0005	0.0005
E_L_ (mV)	-65	-65	-65	-65
E_Na_ (mV)	50	50	50	50
E_K_ (mV)	-80	-80	-80	-80

c_m_: membrane capacity, R_m_: membrane resistivity, R_a_: intracellular resistivity, E_L_: equilibrium potential of the leak current, E_Na_: equilibrium potential of the sodium current, E_k_: equilibrium potential of the K^+^ current.

As suggested by the CA3 models built earlier [24,31,36,37] and anatomical studies [21,38], each pyramidal cell receives somatic synaptic inhibition from the basket cell, distal apical inhibition from the OLM cell and medial dendritic excitation from the other pyramidal cell. It has been reported that the input from the DG projects proximally to the stratum lucidum, whereas the input from the EC projects distally to the stratum lacunosum-moleculare [36]. The input from the DG was relatively selective and projected to some of the CA3 pyramidal cells [36]. Thus, in our model, one pyramidal cell receives proximal excitation from the DG and both pyramidal cells receive distal excitation from the EC.

### Basket cell

The simulated basket cell has one compartment [31,36,37] and contains leak current, sodium current, and delayed rectifier K^+^ current [31,53]. It is the same as the basket cell model built by Neymotin et al. [31]. It has been proven that basket cells are prevalent within the CA3 region [36], and they are fast-spiking interneurons [53]. With fast kinetics of the sodium current and delayed rectifier K^+^ current, the basket cell has the ability to fire repetitive spikes at high frequencies (Figure 5B). As a consequence, compared to the OLM cell, the basket cell affects pyramidal cells with a higher frequency. The basket cell inhibits the somata of both pyramidal cells directly and simultaneously, which help to control the synchrony of action potentials of pyramidal cells [46]. The parameters of ionic conductance used in the model are listed in Table 2. According to the CA3 models built earlier [36,37] and anatomical studies [38], the basket cell receives excitatory inputs from pyramidal cells and the inhibitory input from itself.

**Table 2:** Passive parameters and ionic conductance of channels for all compartments of basket and OLM cells

**Mechanism**	**Basket cell**	**OLM cell**
C_m,_ μF/cm^2^	1	1
R_m,_ Ωcm^2^	60000	60000
Ra_,_ Ωcm	200	200
Leak conductance [S/cm^2^]	0.000017	0.000017
Sodium conductance [S/cm^2^]	0.035	0.035
Delayed rectifier K^+^ conductance [S/cm^2^]	0.0009	0.0009
Ca^2+^-activated K^+^ conductance [S/cm^2^]	–	0.01
Ca^2+^-activated [S/cm^2^]	–	0.001
I_h_ conductance [S/cm^2^]	–	0.15
E_L_ (mV)	-65	-65
E_Na_ (mV)	66	55
E_K_ (mV)	-90	-90
E_Ca_ (mV)	–	120
E_h_ (mV)	–	-40

c_m_: membrane capacity, R_m_: membrane resistivity, R_a_: intracellular resistivity, E_L_: equilibrium potential of the leak current, E_Na_: equilibrium potential of the sodium current, E_k_: equilibrium potential of the K^+^ current, E_Ca_: equilibrium potential of the Ca^2+^ current, E_h_: equilibrium potential of the h current.

### OLM cell

The simulated OLM cell has one compartment [31,36,37] and contains leak current, sodium current, delayed rectifier K^+^ current, Ca^2+^-activated K^+^ current, Ca^2+^ current, and hyperpolarization-activated current I_h_[31,54]. The Ca^2+^-activated K^+^ current allows the cells to have long lasting inactivation after bursting [31], and as well as Ca^2+^ current, help the cells to produce spike-frequency adaptation [54]. The OLM cell also has hyperpolarization-activated current I_h_ for bursting [31]. The OLM cell projects to the dendrites of both pyramidal cells in the model, and affects the membrane potentials there. As glutamatergic receptors locate in the dendrites of pyramidal cells and their conductance is related to the local membrane potentials [47,48], the OLM cell controls the efficacy of glutamatergic receptors, which affects Mg^2+^-free-ACSF induced epileptiform activities. The parameters of ionic conductance used in the model are listed in Table 2, which are the same as the parameters used by Wang [54]. According to anatomical studies, the OLM cell receives excitatory inputs from pyramidal cells and the inhibitory input from the basket cell [37,38].

### Synaptic properties

In this model, AMPA, NMDA and GABA_A_ receptors are considered, which are the commonest receptors in CA3 models [31,35]. As the network built here is small, AMPA and NMDA receptors are present in all excitatory connections, and GABA_A_ receptors are present in all inhibitory connections. AMPA and GABA_A_ receptors are modelled by a standard NEURON double-exponential mechanism [47,48]. The synaptic conductance g_syn_(t) is given by:

(1) $$g_{\mathrm{syn}}\left(t\right)=c*\left[\exp\;\left(-\frac{t}{\tau_{2}}\right)-\exp\;\left(-\frac{t}{\tau_{1}}\right)\right]$$

where c is the weight, τ_1_ is the rising time constant, and τ_2_ is the falling time constant. The NMDA receptor conductance is dependent on the local membrane potential and the external Mg^2+^ concentration [31,47], and it is given by:

(2) $$g_{\mathrm{syn}}\left(t\right)=c*\frac{\exp\;\left(-\frac{t}{\tau_{2}}\right)-\exp\;\left(-\frac{t}{\tau_{1}}\right)}{1+\frac{\mathrm{Mg}}{3.57}\exp\;\left(-0.062V\left(t\right)\right)}$$

where Mg is the external Mg^2+^ concentration in mM, V(t) is the local membrane potential in mV, and the other variables are as in eq. (1). AMPA and NMDA receptors have reversal potentials of 0 mV, while GABA_A_ receptors have reversal potentials of -80 mV [31]. The parameters of synapses are present in Table 3, which are based on the model built by Neymotin et al. [31].

**Table 3:** Synaptic parameters

**Presynaptic**	**Postsynaptic**	**Receptor**	**T**_**1**_**(ms)**	**T**_**2**_**(ms)**	**Conductance (μS)**
Pyramidal	Pyramidal	AMPA	0.05	15.3	0.00045
Pyramidal	Pyramidal	NMDA	15	150	0.00036
Pyramidal	Basket	AMPA	0.05	5.3	0.18
Pyramidal	Basket	NMDA	15	150	0.36
Pyramidal	OLM	AMPA	0.05	5.3	0.18
Pyramidal	OLM	AMPA	15	150	0.36
Basket	Pyramidal	GABA_A_	0.07	9.1	0.13
Basket	Basket	GABA_A_	0.07	9.1	0.65
Basket	OLM	GABA_A_	20	40	0.39
OLM	Pyramidal	GABA_A_	0.2	20	1.3
DG	Pyramidal	AMPA	0.05	5.3	0.0036
DG	Pyramidal	NMDA	15	150	0.0012
EC	Pyramidal	AMPA	0.05	5.3	0.00036
EC	Pyramidal	NMDA	15	150	0.000072

### Model inputs

The model receives two extrahippocampal inputs from the DG and EC respectively. The input from the DG projects to the proximal dendrites of pyramidal cells, whereas the input from the EC projects distally to the dendrites of pyramidal cells [55]. In the simulation, the input from the DG was relatively strong and selective, exciting only one of the pyramidal cells. However, the input from the EC was relatively weak and diffuse, exciting both pyramidal cells [21,36,55]. As suggested by the data from experiments, interictal discharges were induced by Mg^2+^-free-ACSF in hippocampal slices, and CA3 were considered to be the initiation place [16,18,19]. As hippocampal slices didn’t contain EC, we only considered the input from the DG, which followed a Possion process (λ = 1) in the model [31]. In combined EC-hippocampal slices, alternate interictal and ictal discharges were induced by Mg^2+^-free-ACSF, and epileptiform activities in the EC and DG were synchronized with each other [18,19]. An extral pyramidal cell was built, which received current injections and generated ictal-like and interictal-like discharges, to simulate the input signals from the DG and EC. AMPA and NMDA receptors were presented in all the connections, and the connection strength between the input from the DG and the model was larger than that between the input from the EC and the model.

## Appendix

### Pyramidal cell

The somatic (s), proximal dendritic (pd), medium dendritic (md), and distal dendritic (dd) compartments obey the following current balance equations:

$$\begin{array}{l} c\frac{{\mathrm{dV}}_{s}}{\mathrm{dt}}=-I_{L}-I_{\mathrm{Na}}-I_{\mathrm{kdr}}-I_{\mathrm{kc}}-I_{\mathrm{AHP}}-I_{\mathrm{km}}-I_{\mathrm{CaL}}-I_{\mathrm{CaN}}-I_{\mathrm{CaT}}-I_{\mathrm{syn}}\\ c\frac{{\mathrm{dV}}_{\mathrm{pd}}}{\mathrm{dt}}=-I_{L}-I_{\mathrm{Na}}-I_{\mathrm{kdr}}-I_{\mathrm{kc}}-I_{\mathrm{km}}-I_{\mathrm{CaL}}-I_{\mathrm{CaN}}-I_{\mathrm{CaT}}-I_{\mathrm{syn}}\\ c\frac{{\mathrm{dV}}_{\mathrm{md}}}{\mathrm{dt}}=-I_{L}-I_{\mathrm{Na}}-I_{\mathrm{kdr}}-I_{\mathrm{kc}}-I_{\mathrm{AHP}}-I_{\mathrm{km}}-I_{\mathrm{CaN}}-I_{\mathrm{CaT}}-I_{\mathrm{syn}}\\ c\frac{{\mathrm{dV}}_{\mathrm{dd}}}{\mathrm{dt}}=-I_{L}-I_{\mathrm{Na}}-I_{\mathrm{kdr}}-I_{\mathrm{AHP}}-I_{\mathrm{km}}-I_{\mathrm{CaN}}-I_{\mathrm{CaT}}-I_{\mathrm{syn}} \end{array}$$

Where I_L_ is the leak current, I_Na_ is the sodium current, I_kdr_ is the delayed rectifier K^+^ current, I_kc_ is the Ca^2+^ activated K^+^ current, I_AHP_ is the AHP K^+^ current, I_km_ is the M-type K^+^ current, I_CaL_, I_CaN_, and I_CaT_ are the L-, N- and T-type Ca^2+^ currents, and I_syn_ is the synaptic current. The conductance and reversal potential values for all ionic currents are listed in Table 1.

The sodium current is described by [56]:

$$\begin{array}{l} I_{\mathrm{Na}}=g_{\mathrm{Na}}m^{3}h\left(V-E_{\mathrm{Na}}\right)\\ \alpha_{m}=\frac{0.32\left(13.1-V\right)}{\exp\;\left(\frac{13.1-V}{4}\right)-1}\\ \beta_{m}=\frac{0.28\left(V-40.1\right)}{\exp\;\left(\frac{V-40.1}{5}\right)-1}\\ \alpha_{h}=0.128\;\exp\;\left(\frac{17-V}{18}\right)\\ \beta_{h}=\frac{4}{1+\exp\;\left(\frac{40-V}{5}\right)} \end{array}$$

The K^+^ currents are given by [39]:

$$\begin{array}{l} I_{\mathrm{kdr}}=g_{\mathrm{kdr}}m^{3}h\left(V-E_{k}\right)\\ \alpha_{m}=0.03\exp\;\left[2\left(V+32\right)*\frac{F}{\mathrm{RT}}\right]\\ \beta_{m}=0.03\exp\;\left[3\left(V+32\right)*\frac{F}{\mathrm{RT}}\right]\\ \alpha_{h}=0.001\exp\;\left[-2\left(V+61\right)*\frac{F}{\mathrm{RT}}\right]\\ \beta_{h}=0.001\\ I_{\mathrm{km}}=g_{\mathrm{km}}m\left(V-E_{k}\right)\\ \alpha_{m}=0.006\exp\;\left[0.6\left(V+55\right)*\frac{F}{\mathrm{RT}}\right]\\ \beta_{m}=0.06\exp\;\left[-9.4\left(V+55\right)*\frac{F}{\mathrm{RT}}\right]\\ I_{\mathrm{AHP}}=g_{\mathrm{AHP}}m\left(V-E_{k}\right)\\ \alpha_{m}=1.3*10^{13}*{\left[{\mathrm{Ca}}^{2+}\right]}_{i}^{4}\\ \beta_{m}=0.005\\ I_{\mathrm{kc}}=g_{\mathrm{kc}}m\left(V-E_{k}\right)\\ \alpha_{m}=\frac{0.28{\left[{\mathrm{Ca}}^{2+}\right]}_{i}}{{\left[{\mathrm{Ca}}^{2+}\right]}_{i}+0.48*10^{-3}*\exp\;\left(-1.68V*\frac{F}{\mathrm{RT}}\right)}\\ \beta_{m}=\frac{0.48}{1+\frac{{\left[{\mathrm{Ca}}^{2+}\right]}_{i}}{0.13*10^{-6}*\exp\;\left(-2V*\frac{F}{\mathrm{RT}}\right)}} \end{array}$$

The Ca^2+^ currents are given by [57]:

$$\begin{array}{l} I_{\mathrm{CaL}}=-g_{\mathrm{CaL}}m^{2}V\frac{1-\frac{{\left[{\mathrm{Ca}}^{2+}\right]}_{i}}{{\left[{\mathrm{Ca}}^{2+}\right]}_{o}}\exp\;\left(2V*\frac{F}{\mathrm{kT}}\right)}{1-\exp\;\left(2V*\frac{F}{\mathrm{kT}}\right)}\\ \alpha_{m}=\frac{15.69\left(81.5-V\right)}{\exp\;\left(\frac{81.5-V}{10}\right)-1}\\ \beta_{m}=0.29\exp\;\left(\frac{-V}{10.86}\right)\\ I_{\mathrm{CaN}}=-g_{\mathrm{CaN}}m^{2}\mathrm{hV}\frac{1-\frac{{\left[{\mathrm{Ca}}^{2+}\right]}_{i}}{{\left[{\mathrm{Ca}}^{2+}\right]}_{o}}\exp\;\left(2V*\frac{F}{\mathrm{kT}}\right)}{1-\exp\;\left(2V*\frac{F}{\mathrm{kT}}\right)}\\ \alpha_{m}=\frac{0.19\left(19.88-V\right)}{\exp\;\left(\frac{19.88-V}{10}\right)-1}\\ \beta_{m}=0.046\exp\;\left(\frac{-V}{20.73}\right)\\ \alpha_{h}=1.6*10^{-4}\exp\;\left(\frac{-V}{48.4}\right)\\ \beta_{h}=\frac{1}{\exp\;\left(\frac{39-V}{10}\right)+1}\\ I_{\mathrm{CaT}}=-g_{\mathrm{CaT}}m^{2}\mathrm{hV}\frac{1-\frac{{\left[{\mathrm{Ca}}^{2+}\right]}_{i}}{{\left[{\mathrm{Ca}}^{2+}\right]}_{o}}\exp\;\left(2V*\frac{F}{\mathrm{kT}}\right)}{1-\exp\;\left(2V*\frac{F}{\mathrm{kT}}\right)}\\ \alpha_{m}=\frac{0.2\left(19.26-V\right)}{\exp\;\left(\frac{19.26-V}{10}\right)-1}\\ \beta_{m}=0.009\exp\;\left(\frac{-V}{22.03}\right)\\ \alpha_{h}=1.0*10^{-6}\exp\;\left(\frac{-V}{16.26}\right)\\ \beta_{h}=\frac{1}{\exp\;\left(\frac{29.79-V}{10}\right)+1} \end{array}$$

Where [Ca^2+^]_o_ = 2 mM, resting [Ca^2+^]_i_ = 50 nM, and the dynamics of [Ca^2+^]_i_ come from Migliore et al. [39].

In all the equations above, V is the membrane potential, k is the Boltzmann’s constant, F is the Faraday’s constant, T is the absolute temperature, R is the gas constant, [Ca^2+^]_i_ is the intracellular Ca^2+^ concentration, and [Ca^2+^]_o_ is the extracellular Ca^2+^ concentration. State variables m and h obey the following equations:

$$\begin{array}{l} \frac{\mathrm{dm}}{\mathrm{dt}}=\alpha_{m}\left(1-m\right)-\beta_{m}m\\ \frac{\mathrm{dh}}{\mathrm{dt}}=\alpha_{h}\left(1-h\right)-\beta_{h}h \end{array}$$

### Basket cell

The somatic compartment obeys the following current balance equation:

$$c\frac{\mathrm{dV}}{\mathrm{dt}}=-I_{L}-I_{\mathrm{Na}}-I_{\mathrm{kdr}}-I_{\mathrm{syn}}$$

Where I_L_ is the leak current, I_Na_ is the sodium current,  I_kdr_ is the delayed rectifier K^+^ current, and I_syn_ is the synaptic current. The conductance and reversal potential values for all ionic currents are listed in Table 2. The model built here is the same as that built by Neymotin et al. [31].

The sodium current is described by:

$$\begin{array}{l} I_{\mathrm{Na}}=g_{\mathrm{Na}}m^{3}h\left(V-E_{\mathrm{Na}}\right)\\ \alpha_{m}=-\frac{0.1\left(V+35\right)}{\exp\;\left(-0.1\left(V+35\right)\right)-1}\\ \beta_{m}=4\exp\;\left(-\frac{V+60}{18}\right)\\ \alpha_{h}=0.35\exp\;\left(-\frac{V+58}{20}\right)\\ \beta_{h}=\frac{5}{\exp\;\left(-0.1\left(V+28\right)\right)+1} \end{array}$$

The delayed rectifier K^+^ current is given by:

$$\begin{array}{l} I_{\mathrm{kdr}}=g_{\mathrm{kdr}}m^{4}\left(V-E_{k}\right)\\ \alpha_{m}=-0.05\left(V+34\right)/(\exp\;\left(-0.1\left(V+34\right)\right)-1)\\ \beta_{m}=0.625\exp\;\left(-\frac{V+44}{80}\right) \end{array}$$

In all the equations above, V is the membrane potential, State variables m and h obey the following equations:

$$\begin{array}{l} \frac{\mathrm{dm}}{\mathrm{dt}}=\alpha_{m}\left(1-m\right)-\beta_{m}m\\ \frac{\mathrm{dh}}{\mathrm{dt}}=\alpha_{h}\left(1-h\right)-\beta_{h}h \end{array}$$

### OLM cell

The somatic compartment obeys the following current balance equation:

$$c\frac{\mathrm{dV}}{\mathrm{dt}}=-I_{L}-I_{\mathrm{Na}}-I_{\mathrm{kdr}}-I_{h}-I_{\mathrm{Ca}}-I_{\mathrm{kc}}-I_{\mathrm{syn}}$$

Where I_L_ is the leak current, I_Na_ is the sodium current, I_kdr_ is the delayed rectifier K^+^ current, I_h_ is the hyperpolarization-activated current, I_Ca_ is the Ca^2+^ current, I_kc_ is the Ca^2+^ activated K^+^ current, and I_syn_ is the synaptic current. In this model, the sodium current I_Na_ and the delayed rectifier K^+^ current I_kdr_ are the same as in the basket cell. The conductance and reversal potential values for all ionic currents are listed in Table 2. The model built here is the same as that built by Wang [54].

The h current is described by:

$$\begin{array}{l} I_{h}=g_{h}n\left(V-E_{h}\right)\\ n_{\infty }=1/\left(1+\exp\;\left(\frac{V+80}{10}\right)\right)\\ \tau_{n}=\frac{200}{\exp\;\left(\frac{V+70}{20}\right)+\exp\;\left(-\frac{V+70}{20}\right)}+5\\ n_{\infty }=\frac{\alpha_{n}}{\alpha_{n}+\beta_{n}}\\ \tau_{n}=\frac{1}{\alpha_{n}+\beta_{n}}\\ \frac{\mathrm{dn}}{\mathrm{dt}}=\alpha_{n}\left(1-n\right)-\beta_{n}n \end{array}$$

The Ca^2+^ current is given by:

$$\begin{array}{l} I_{\mathrm{Ca}}=g_{\mathrm{Ca}}m_{\infty }^{2}\left(V-E_{\mathrm{Ca}}\right)\\ m_{\infty }=1/\left(1+\exp\;\left(-\frac{\left(V+20\right)}{9}\right)\right) \end{array}$$

Where m is replaced by its steady-state m_*∞*__._

The Ca^2+^ activated K^+^ current is given by:

$$\begin{array}{l} I_{\mathrm{kc}}=g_{\mathrm{kc}}\frac{{\left[{\mathrm{Ca}}^{2+}\right]}_{i}}{{\left[{\mathrm{Ca}}^{2+}\right]}_{i}+30}\left(V-E_{k}\right)\\ \frac{d{\left[{\mathrm{Ca}}^{2+}\right]}_{i}}{\mathrm{dt}}=-0.002I_{\mathrm{Ca}}-{\left[{\mathrm{Ca}}^{2+}\right]}_{i}/80 \end{array}$$

In all the equations above, V is the membrane potential, [Ca^2+^]_i_ is the intracellular Ca^2+^ concentration.

## Competing interests

The authors declare that they have no competing interests.

## Authors’ contributions

HR built the model and drafted the manuscript, YJS did the experiments, PMZ and QCL instructed the experiments, PMZ and PJL improved the model, PMZ improved the manuscript. All authors read and approved the final manuscript.
